# Case of Hydrophobia

**Published:** 1814-02

**Authors:** 


*For the Medical and Physical Journal.*

### *Case of Hydrophobia.*

HOWEVER sanguine may have been the hopes that some individuals have lately entertained of the sanative effects of blood-letting in hydrophobia, sanctioned by the authority of Dr. Schoolbred in the East, and of some practitioners in this country, the following case related in the Madras Courier, Jan. 5, 1813, as far as a solitary instance can have weight, is decidedly against the practice. It may be objected indeed that a newspaper is not very good authority, and that the names of the practitioners are concealed. Such an objection is certainly strong, yet the case is so circumstantially narrated, and evidently in the language of a medical reporter, that there is little cause for hesitation in admitting its authenticity.

The dog, which it seems had previously exhibited no marked signs of deviation from health, bit three individuals on the 2d of May, only one of whom, Miss D. afterwards became affected with hydrophobia. The bitten parts were cut out, and mercury was exhibited so as to occasion salivation; yet on the 14th of the following June, the disease made its appearance, the particulars of which are thus recorded in the Madras Courier.

"This

"This day (14th of June), she complained of great languor; in the evening she took some camomile tea, which operated powerfully as an emetic. She perspired profusely during the greater part of the night. On the 15th she came to the breakfast table, but had no appetite, and eat nothing. When water was brought to her to wash her mouth, she was observed to tremble. About noon some arrow-root was dressed, which she requested should be given to her in a coffee cup, and not in a glass, as her friends intended; she shivered strongly when she saw the arrow-root. She succeeded, however, in swallowing a little of it, although with infinite difficulty. At dinner she eat a little, but now very evidently shuddered when water was brought to her view. She swallowed a little tea, and ate a small portion of bread, about five P. M. At seven swallowed a little coffee; in an hour after she perspired very profusely, and complained of great giddiness. Between nine and ten o'clock the doors of her room were opened to admit more air; she instantly complained of the current of air giving her great uneasiness, and begged to be removed from it. She often attempted to sleep during night, but was almost instantly awakened by frightful dreams, hastily starting up, trembling, and shivering. About three A. M. she complained of a sensation of extreme heat in her throat and belly; the giddiness at the same time continued unabated. At her own desire, water was brought to her bed-side to bathe her feet; on her perceiving it she shuddered strongly, and turned from it as if terrified. In a short time, she so far conquered her aversion to water, as to place her feet in the tub for a few minutes; for a little time she thought the giddiness relieved. I was called to visit her on the morning of the 16th, and reached her house about half-past seven o'clock, where I was soon joined by two other physicians: we found her sitting on a bed in a reclining position, supported by pillows, and her arms placed across her body. The muscles of the face were frequently convulsed, and the angles of the mouth occasionally drawn back. Her eyes were glassy, and at intervals fixed, with a wild unmeaning stare. The collection of saliva in the mouth gave great uneasiness, although it was partially expelled by expiration, in a state of foam. Every three or four minutes the viscid phlegm seemed to irritate and throw into convulsions the muscles of the throat, when she exerted her whole force to expel the offending fluid.

"The skin was covered with perspiration; it ran over the face in drops—her pulse was about 100, and sharp—her respiration was hurried, and unequal—the countenance expressed great anxiety, occasionally it indicated extreme

 agony.

agony. She sometimes glanced her eyes through the room, while her countenance expressed a suspicious watchful dread of some impending evil. Except when the irritation and spasms of the muscles of deglutition occurred, the chief uneasiness she complained of, was a painful sense of heat in the throat; the giddiness of the head also continued to trouble her. In general, she seemed unwilling to speak, and when she attempted it, the tongue faultered, the lips trembled, and her lower jaw did not readily obey the will. Her speech was consequently hurried, and not always very intelligible. She seemed sensible, and perfectly conscious of what was passing around her: she expressed an earnest desire, that her father and sister should remain constantly by her. At her own request, a little rice was brought to her, of which she swallowed a small portion, but after an extremely painful exertion of the muscles of the throat. On hearing one of us express a wish to see water brought into her view, she readily complied, and showed great willingness to gratify us, although she was evidently afraid to make the trial. She even put a cup, which contained some water, to her lips, and attempted to swallow a little, which was instantly rejected with great force by the spasmodic constriction of the muscles of deglutition. This attempt was also accompanied with general convulsions. When water was brought in her view, a short time after, she turned from it with a countenance expressive of horror.

"When the hand was examined, it was found that the wound was perfectly healed, leaving a slight eschar in the skin, but without induration or tumefaction of the parts under it.

"She was bled in the right arm by a large orifice, and the blood permitted to flow until faintness was produced; the quantity of blood taken away was about twenty-four ounces. Soon after the vein was opened, she very intelligibly expressed herself generally relieved, particularly of a heaviness and giddiness of the head. The spasms of the muscles of the throat were evidently less violent, and also much less frequent; her countenance became comparatively placid and natural. Before the vein was closed, the pulse was so weak as scarcely to be felt at the wrist. During her state of faintishness, a phial containing eau de luce was applied to her nose, which instantly produced strong involuntary actions of the muscles of the throat and face.

"In about ten or fifteen minutes after the arm was tied up, the morbid symptoms returned with increased violence; and debility supervened rapidly. An enema, with four drachms of the tinct. of opium, was now exhibited. Shortly after, after, she was suddenly seized with a severe pain and sensation of burning in the regions of the epigastrium and umbilicus, in consequence of which she shrieked out, in great agony. She continued to scream for about twenty minutes, and then became suddenly quiet. A warm bath having been previously prepared, she was now put into it: she expressed no horror at the sight of the water, nor on immersion. After being removed from the bath, she was put to bed. The system now seemed extremely relaxed and debilitated: the pulse could not be felt at the wrist; the pupils of the eyes were dilated; the countenance was sunk; and the skin covered with a cold perspiration: foam was no longer formed at the mouth, probably the power of swallowing returned. Her breathing was hurried, and accompanied with a convulsive sob. At a quarter past nine A. M. she expired.

"The history of this case confirms the melancholy fact, that the bite of a dog, apparently healthy, is occasionally followed by hydrophobic symptoms. While the animal who inflicts the wound continues to eat, and particularly to drink, and when its temper is not much altered, the only certain means of preventing this disease is too generally deemed premature; the friends of the patient are lulled into security, and readily believe, because they anxiously hope, that the dog is not affected with one of the most destructive of all maladies. We may likewise learn, that complete excision of the lacerated parts, two days after the infliction of the wounds, is not likely to be generally successful. In this case, the wound being situated in a muscular part, an excellent opportunity was afforded of removing the torn integuments and muscles, more effectually than generally occurs in wounds of this kind.

"Mercury has been recommended, both as a preventive and cure of this disease, by many respectable names; although it was here carried to a very considerable extent, and exhibited early, the supervention of the disease was not prevented by it; probably the symptoms were not in the least protracted."

